# Association of Lipoprotein(a) With Atherosclerotic Plaque Progression

**DOI:** 10.1016/j.jacc.2021.10.044

**Published:** 2022-01-25

**Authors:** Yannick Kaiser, Marwa Daghem, Evangelos Tzolos, Mohammed N. Meah, Mhairi K. Doris, Alistair J. Moss, Jacek Kwiecinski, Jeffrey Kroon, Nick S. Nurmohamed, Pim van der Harst, Philip D. Adamson, Michelle C. Williams, Damini Dey, David E. Newby, Erik S.G. Stroes, Kang H. Zheng, Marc R. Dweck

**Affiliations:** a Department of Vascular Medicine, Amsterdam Cardiovascular Sciences, Amsterdam UMC, University of Amsterdam, Amsterdam, the Netherlands; b British Heart Foundation Centre for Cardiovascular Science, University of Edinburgh, United Kingdom; c Department of Cardiovascular Science, National Institute of Health Research Biomedical Research Centre Leicester, University of Leicester, Leicester, United Kingdom; d Department of Interventional Cardiology and Angiology, Institute of Cardiology, Warsaw, Poland; e Department of Experimental Vascular Medicine, Amsterdam Cardiovascular Sciences, Amsterdam UMC, University of Amsterdam, Amsterdam, the Netherlands; f Department of Cardiology, Amsterdam Cardiovascular Sciences, Amsterdam UMC, Vrije Universiteit, Amsterdam, the Netherlands; g Department of Cardiology, University Medical Center Utrecht, Utrecht, the Netherlands; h Christchurch Heart Institute, University of Otago, Christchurch, New Zealand; i Biomedical Imaging Research Institute, Cedars-Sinai Medical Center, Los Angeles, California, USA

**Keywords:** lipoprotein(a), coronary computed tomography angiography, low-attenuation plaque, ASCVD, atherosclerotic cardiovascular disease, CCTA, coronary computed tomography angiography, LDL, low-density lipoprotein, Lp(a), lipoprotein(a), OxPL, oxidized phospholipids

## Abstract

**Background:**

Lipoprotein(a) [Lp(a)] is associated with increased risk of myocardial infarction, although the mechanism for this observation remains uncertain.

**Objectives:**

This study aims to investigate whether Lp(a) is associated with adverse plaque progression.

**Methods:**

Lp(a) was measured in patients with advanced stable coronary artery disease undergoing coronary computed tomography angiography at baseline and 12 months to assess progression of total, calcific, noncalcific, and low-attenuation plaque (necrotic core) in particular. High Lp(a) was defined as Lp(a) ≥ 70 mg/dL. The relationship of Lp(a) with plaque progression was assessed using linear regression analysis, adjusting for body mass index, segment involvement score, and ASSIGN score (a Scottish cardiovascular risk score comprised of age, sex, smoking, blood pressure, total and high-density lipoprotein [HDL]–cholesterol, diabetes, rheumatoid arthritis, and deprivation index).

**Results:**

A total of 191 patients (65.9 ± 8.3 years of age; 152 [80%] male) were included in the analysis, with median Lp(a) values of 100 (range: 82 to 115) mg/dL and 10 (range: 5 to 24) mg/dL in the high and low Lp(a) groups, respectively. At baseline, there was no difference in coronary artery disease severity or plaque burden. Patients with high Lp(a) showed accelerated progression of low-attenuation plaque compared with low Lp(a) patients (26.2 ± 88.4 mm^3^ vs −0.7 ± 50.1 mm^3^; *P* = 0.020). Multivariable linear regression analysis confirmed the relation between Lp(a) and low-attenuation plaque volume progression (β = 10.5% increase for each 50 mg/dL Lp(a), 95% CI: 0.7%-20.3%). There was no difference in total, calcific, and noncalcific plaque volume progression.

**Conclusions:**

Among patients with advanced stable coronary artery disease, Lp(a) is associated with accelerated progression of coronary low-attenuation plaque (necrotic core). This may explain the association between Lp(a) and the high residual risk of myocardial infarction, providing support for Lp(a) as a treatment target in atherosclerosis.

Lipoprotein(a) (Lp[a]) has been widely recognized as a prevalent and independent cardiovascular risk factor. Post hoc analyses of recent clinical trials have substantiated Lp(a) as a potent marker of residual cardiovascular risk, even in patients receiving intensive lipid-lowering therapy (1). Current guidelines recommend measuring Lp(a) to identify patients with a high lifetime risk for atherosclerotic cardiovascular disease (ASCVD) (2). Although outcome studies of therapies dedicated to specific lowering of Lp(a) are ongoing (Assessing the impact of Lipoprotein(a) Lowering With TQJ230 on Major Cardiovascular Events in Patients With CVD [Lp(a) HORIZON]; NCT04023552), the exact mechanisms underlying the increased ASCVD-risk mediated by Lp(a) remain a matter of debate (3).

Structurally, Lp(a) is a low-density lipoprotein (LDL) to which apolipoprotein(a) is covalently bound; the latter carrying proinflammatory oxidized phospholipids (OxPLs) (4). The atherogenic mechanisms of this multifaceted particle are thought to include accumulation of the LDL component in atherosclerotic plaque, prothrombotic effects due to interference of the apolipoprotein(a) tail with plasminogen activation, as well as induction of a multilevel proinflammatory response mediated by OxPL-cargo (5). The prothrombotic and proinflammatory effects of Lp(a) have been suggested to promote plaque destabilization leading to plaque rupture and atherothrombotic events (6). Previous studies have shown an association between high serum levels of Lp(a) and high baseline atherosclerotic plaque volumes and the presence of adverse plaque features in patients with coronary artery disease (7,8). However, data on the interaction between Lp(a) and progression of coronary plaque volumes and composition in contemporary patients are lacking.

Advanced quantitative plaque assessments on coronary computed tomography angiography (CCTA) now allow the assessment of both calcific and noncalcific atherosclerotic plaque types facilitating the tracking of disease progression as well as changes in coronary plaque morphology with good reproducibility (9). Moreover, recent studies have shown a strong association between plaque composition determined by quantitative computed tomography (CT) analysis and clinical events (10,11). In particular, the burden of low-attenuation plaque (attenuation density <30 HU), which serves as a marker of necrotic core, provides powerful prediction of future myocardial infarction outperforming clinical risk scores, severity of luminal stenosis, and CT calcium scoring (12).

In this study, we assessed whether high concentrations of serum Lp(a) are associated with progression of adverse plaque phenotype in a cohort of patients with advanced stable coronary artery disease who were already using guideline-directed preventative therapies.

## Methods

### Study design

Participants were recruited as part of the DIAMOND (Dual Antiplatelet Therapy to Inhibit Coronary Atherosclerosis and Myocardial Injury in Patients With Necrotic High-Risk Coronary Plaque Disease) study, a double-blind, randomized, parallel-group, placebo-controlled trial conducted at a single center in Edinburgh, United Kingdom (13). The primary results of this study have previously been published and showed that dual antiplatelet therapy did not impact coronary atherosclerotic disease progression or adverse plaque phenotype. The study was approved by the local Institutional Review Board, the Scottish Research Ethics Committee (REC reference: 14/SS/0089), the Medicines and Healthcare products Regulatory Agency, and the United Kingdom Administration of Radiation Substances Advisory Committee. It was performed in accordance with the Declaration of Helsinki. All patients provided written informed consent before any study procedures were initiated. The data that support the findings of this study are available from the corresponding author upon reasonable request.

### Patient selection

Patients with clinically stable multivessel coronary artery disease were recruited prospectively from the Edinburgh Heart Centre, United Kingdom, between March 2015 and March 2017. Patients were included if aged older than 40 years and had evidence of angiographically proven multivessel coronary artery disease, defined as at least 2 major epicardial vessels with any combination of either >50% luminal stenosis or previous revascularization (percutaneous coronary intervention or coronary artery bypass graft surgery). Patients were excluded in the event of coronary revascularization within the preceding 3 months or acute coronary syndrome within the previous 12 months.

### Laboratory measurements

Baseline serum and plasma samples were obtained at the time of recruitment and stored at −80°C until further use. Hematology, biochemistry, and lipid panels were determined according to standardized operating procedures in a core laboratory. Low-density-lipoprotein cholesterol was calculated using the Friedewald equation (14). Lp(a) was measured at a later time point from frozen serum samples, using a KIV_2_-independent immunoassay (Randox Laboratories).

### Image acquisition

Baseline coronary CT scans were acquired on a hybrid positron emission tomography–CT scanner (64-multidetector Biograph mCT, Siemens Medical Systems) using a standardized study protocol. Before scanning, participants with a resting heart rate >65 beats/min were administered oral β-blockade (50 to 100 mg metoprolol) unless contraindicated. An electrocardiogram–gated breath-held noncontrast CT scan (tube voltage, 120 kV; tube current based on body habits) was performed for coronary CT calcium scoring and reconstructed in the axial plane with 3-mm slice width and 1.5-mm increment. Finally, an electrocardiogram-gated coronary CT angiogram (tube voltage 120 kV, tube current based on body habits) was performed in mid-diastole during held expiration following sublingual glyceryl trinitrate. Repeat CCTA and calcium scoring were performed using the same imaging protocol and on the same scanner after an interval of 12 months.

### Image analysis

#### Coronary calcium score

Coronary calcium on noncontrast CT was quantified on both a per-participant and per-segment level by an experienced observer using dedicated software (Vitrea Advanced, Toshiba Systems). Calcification was quantified as calcium score (Agatston units [AU]). Calcium score was derived using the Agatston method (15). Coronary stents were excluded from the per-patient analysis by only including calcium proximal or distal to the border of the stented segment.

#### CCTA image analysis

CCTA segments and vessels were identified by landmarks such as bifurcations and side branches. Segments and vessels with stents were excluded from the analyses and an equal number of segments and vessels were assessed at baseline and follow-up. Segment-wise analysis was performed according to the 17-segment modified American Heart Association classification (15). Coronary atherosclerotic lesions were quantified for luminal stenosis severity by visual estimation. A segment involvement score was calculated as the total number of coronary artery segments exhibiting plaque, irrespective of the degree of luminal stenosis within each segment (minimum = 0; maximum = 16) (16).

#### CT plaque analysis

**Figure 1:**
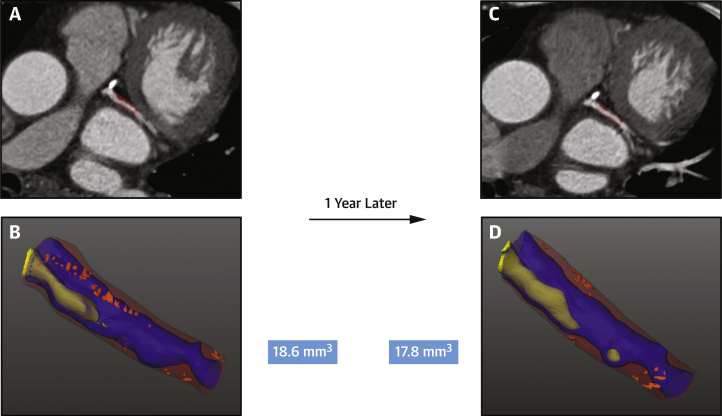
Low-Attenuation Plaque Progression on CCTA in a Patient With Low Lp(a) Coronary computed tomography angiography (CCTA) of a patient with low serum lipoprotein(a) [Lp(a)] concentration (9.2 mg/dL) showing atherosclerotic plaque in the left circumflex artery **(A and C)** with evidence of mixed plaque on automated plaque assessment **(red overlay).** Low-attenuation plaque is visualized in **bright orange** on the 3-dimensional reconstruction **(B and D)** and does not appear to progress on serial scanning (baseline volume 18.6 mm^3^, 1-year volume 17.8 mm^3^).

Plaque measurements were performed using previously validated semiautomated software version 2.5 (AutoPlaque) (Figure 1) by a trained observer blinded to the patient’s clinical status (17). We have previously shown excellent reproducibility for these measurements (9).

Coronary artery centerlines were extracted in a semiautomated fashion for each major artery and any tributary of >2-mm diameter with visually observed disease. A region of interest was placed in the aorta to define blood pool attenuation. Coronary artery segments were defined manually according to Society of Cardiovascular Computed Tomography guidance (18). All suitable vessel segments were manually identified and vessel wall and plaque constituents were automatically determined using scan-specific thresholds with manual adjustments made as required. Segments with coronary stents were excluded from the analysis.

Coronary atherosclerotic plaque volumes were measured for total plaque, calcific and noncalcific plaque, fibro-fatty plaque, and low-attenuation plaque as a marker of necrotic core. After delineating the lumen, the software automatically adjusted the cutoff points for different plaque types based on attenuation measured in the thoracic aorta. Plaque volumes (measured in mm^3^) for each plaque type were measured across all coronary segments and summed to generate the total plaque volume on a per-patient level. Baseline plaque burdens were calculated by dividing plaque volumes by the coronary vessel volume and multiplying by 100. Plaque progression was defined as the difference in plaque volumes between the baseline and the follow-up CCTA scan. We used plaque volume for this progression analysis as this is a less-derived measurement than plaque burden; therefore, it is more suitable for assessing changes in plaque status in individual patients over time.

#### Statistical analysis

Data are presented as mean ± SD for normally distributed variables, median with IQR for non-normally distributed variables, and number (percentage) for categorical variables. Individuals with serum Lp(a) concentrations >70 mg/dL were classified as having high Lp(a), based on the cutoff value for currently ongoing secondary prevention trials (NCT04023552). Between-group comparisons at baseline were assessed using a Student’s *t*-test for normally distributed data, Mann-Whitney *U* for non-normally distributed data, and chi-square or Fisher exact test for categorical data. To investigate the relationship between Lp(a) and plaque progression, we determined the change in plaque volumes from baseline to follow-up. The effect of Lp(a) on the percentage change in the plaque volume was assessed in univariable and in multivariable analysis accounting for body mass index, segment involving score (a prognostic marker of overall plaque burden), and the ASSIGN score (a Scottish cardiovascular risk score comprised of age, sex, smoking, blood pressure, total and high-density lipoprotein [HDL]–cholesterol, diabetes, rheumatoid arthritis, and deprivation index) which incorporates traditional cardiovascular risk factors and is calibrated to the Scottish population (19,20). Given that Lp(a) follows a highly skewed distribution in the general population, but is linearly associated with ASCVD, we standardized the effect size of Lp(a) to a 50-mg/dL increase as previously proposed (21). We constructed further univariable and multivariable models considering the effect of Lp(a) as a categorical variable (high Lp(a) ≥70 mg/dL vs low Lp(a) <70 mg/dL) on the absolute change in plaque volume. A 2-sided *P* < 0.05 was considered statistically significant. The statistical analyses were performed using RStudio software version 4.0.3 (R Foundation for Statistical Computing).

## Results

### Baseline characteristics

A total of 191 individuals in whom Lp(a) was measured and baseline CCTA performed were included in the present analysis. The mean age was 65.3 ± 8.3 years and 153 (79.7%) were male. Patients had advanced stable multivessel coronary atherosclerosis with 138 (71.5%) having a history of prior acute coronary syndrome, 148 (77.9%) having prior percutaneous coronary intervention, and 34 (17.9%) having prior coronary artery bypass graft surgery. At baseline, the use of preventative therapies was high: 180 patients (94.7%) were on statin therapy and 191 (100%) were on antiplatelet therapy. The median serum Lp(a) concentration was 15 mg/dL (IQR: 6 to 58 mg/dL) and the 80th percentile was 78 mg/dL. Across the whole cohort, the baseline total plaque volume was 1,378 mm^3^ (IQR: 1,048 to 1,994 mm^3^) and burden was 58% (IQR: 53% to 6%). Noncalcific plaque volume was 1,282 mm^3^ (IQR: 959 to 1,692 mm^3^) and burden was 54% (IQR: 50% to 58%). Calcific plaque volume was 95 mm^3^ (IQR: 37 to 224 mm^3^) and burden was 3.7% (IQR: 1.7% to 8.5%). Low-attenuation plaque volume was 87 mm^3^ (IQR: 48 to 168 mm^3^) and burden was 3.8% (IQR: 2.2% to 6.3%). Repeat CCTA after 12 months was available for 160 individuals (83.8%).

### Clinical and imaging characteristics in high- and low-Lp(a) groups

There were no differences in body mass index, smoking status, diabetes mellitus, LDL-cholesterol, and serum creatinine concentrations between those with serum Lp(a) concentrations above or below 70 mg/dL (Table 1). Those with Lp(a) ≥70 mg/dL had higher HDL-cholesterol (1.27 ± 0.39 mmol/L vs 1.11 ± 0.28 mmol/L; *P* = 0.003), and lower systolic blood pressure (141 ± 18 mm Hg vs 148 ± 20 mm Hg; *P* = 0.035). The ASSIGN score was also higher in the group with lower Lp(a) concentrations (27.7% ± 16.0% vs 20.6% ± 10.1%; *P* = 0.006).

**Table 1:** Patient Characteristics

	Lipoprotein(a) ≥70 mg/dL (n = 43)	Lipoprotein(a) <70 mg/dL (n = 148)	*P* Value
Baseline			
Lipoprotein(a), mg/dL	100 (82–115)	10 (5–24)	
Clinical			
Age, y	65.0 ± 7.1	65.3 ± 8.7	0.864
Male	31 (72.1)	122 (82.2)	0.217
Body mass index, kg/m^2^	29.1 ± 5.0	30.0 ± 5.2	0.342
Systolic blood pressure, mm Hg	141 ± 18	148 ± 20	**0.035**
Diastolic blood pressure, mm Hg	78 ± 9	82 ± 11	**0.033**
Active smoking	5 (11.6)	23 (15.6)	0.682
Diabetes mellitus	5 (11.6)	31 (21.1)	0.242
Total cholesterol, mmol/L	4.27 ± 0.86	4.18 ± 1.00	0.545
Low-density lipoprotein cholesterol, mmol/L	2.36 ± 0.62	2.22 ± 0.81	0.270
High-density lipoprotein cholesterol, mmol/L	1.27 ± 0.39	1.11 ± 0.28	**0.003**
Triglycerides, mmol/L	1.20 (0.90–1.70)	1.65 (1.20–2.30)	**0.002**
Creatinine, μmol/L	77 ± 10	82 ± 17	0.066
Statin use	43 (100)	137 (93.2)	0.171
Antiplatelet therapy	40 (93)	143 (97.3)	0.399
ASSIGN score	20.6 (10.1)	27.7 (16.0)	**0.006**
Acute coronary syndrome	32 (74.4)	105 (70.9)	0.848
Percutaneous coronary intervention	36 (83.7)	112 (76.2)	0.402
Coronary artery bypass graft	9 (20.9)	25 (17.0)	0.716
Imaging measurements			
Vessel involvement			0.396
0	3 (7.0)	6 (4.1)	
1	8 (18.6)	43 (29.3)	
2	20 (46.5)	64 (43.5)	
3	12 (27.9)	30 (20.4)	
4	0 (0)	4 (2.7)	
Coronary segment involvement score	5.1 (2.3)	5.6 (2.3)	0.192
Coronary calcium score (Agatston units)	378 (137–650)	371 (101–894)	0.902
Total plaque burden, %	57 (54–67)	58 (53–66)	0.531
Calcific plaque burden, %	3.6 (2.2–10.7)	3.9 (1.5–8.5)	0.644
Noncalcific plaque burden, %	53.7 (50.3–60.2)	53.9 (49.6–58.0)	0.584
Low-density plaque burden, %	3.7 (2.1–5.7)	3.8 (2.3–6.5)	0.848
Fibro-fatty plaque burden, %	20.5 (16.0–30.4)	24.3 (16.6–28.8)	0.578
Total plaque volume, mm^3^	1,452 (1,043–1,963)	1,351 (1,061–1,994)	0.997
Calcific plaque volume, mm^3^	98 (38–205)	94 (32–231)	0.857
Noncalcific plaque volume, mm^3^	1,290 (959–1,668)	1,282 (977–1,718)	0.958
Low-density plaque volume, mm^3^	87 (46–144)	87.09 (50–174)	0.742
Fibro-fatty volume, mm^3^	471 (329–388)	571 (313–836)	0.864

Values are mean ± SD, median (IQR), or n (%). **Bold***P* values <0.05 show statistically significant differences.

Coronary artery calcium scores on noncontrast CT were comparable between the high- and low-Lp(a) groups: 378 (IQR: 137 to 650) AU versus 371 (IQR: 101 to 894) AU (*P* = 0.902). On CCTA, there were no baseline differences between high- and low-Lp(a) groups in terms of total, calcific, noncalcific, and low-attenuation plaque volumes (Table 1). Respective plaque burdens were also similar between high- and low-Lp(a) groups (Table 1).

### Accelerated progression of adverse plaque in high-Lp(a) patients

**Figure 2:**
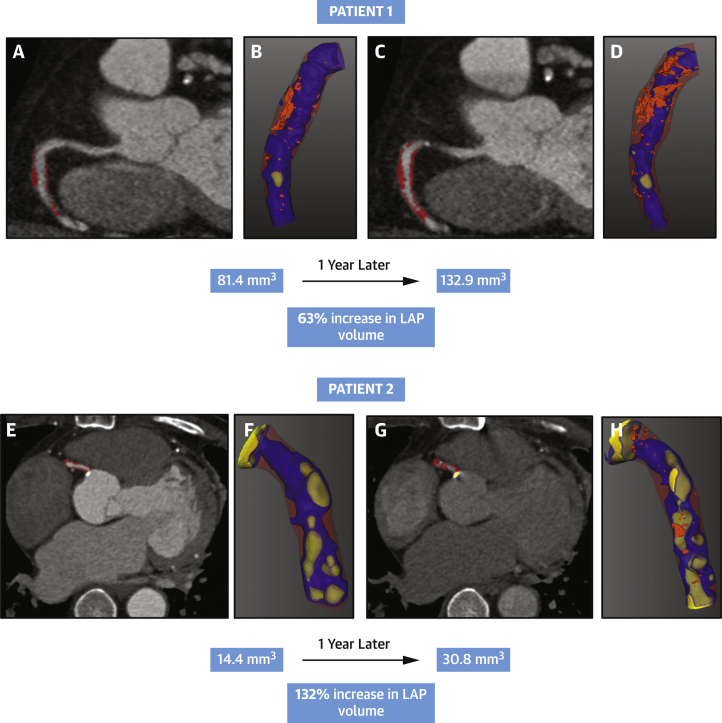
Low-Attenuation Plaque Progression on CCTA in Patients With High Lp(a) CCTA in 2 patients with high serum Lp(a) concentrations (82.2 and 152 mg/dL, respectively). In patient 1, atherosclerotic plaque in the mid-right coronary artery at baseline (**A,** noncalcific highlighted with **red overlay**) and after 1 year **(C).** Low-attenuation plaque is visualized in bright orange on the 3-dimensional reconstructions (**B** and **D**) showing progression from a volume of 81 mm^3^ to 133 mm^3^ 1 year later. Similar representative images are seen in patient 2 with mixed atherosclerotic plaque in the mid-right coronary artery at baseline **(E)** and 1 year **(G).** Low-attenuation plaque progressed on serial scanning from a volume of 14.4 mm^3^**(F)** to 30.8 mm^3^ after 1 year **(H).** LAP = low-attenuation plaque; other abbreviations as in Figure 1.

In the 160 individuals with repeat CCTA, we observed a mean increase of 83.0 ± 293.4 mm^3^ in total plaque volume, 4.4 ± 76.2 mm^3^ in calcific plaque volume, 78.6 ± 274.0 mm^3^ in noncalcific plaque volume, and 4.6 ± 56.7 mm^3^ in low-attenuation plaque volumes. Patients in the high-Lp(a) group showed a larger increase in low-attenuation plaque volumes compared to patients in the low-Lp(a) group (26.2 ± 88.4 mm^3^ vs -0.7 ± 50.1 mm^3^; *P* = 0.020) (Table 2, Figures 1 and 2). Patients in the high-Lp(a) group also showed a larger increase in fibro-fatty plaque volumes compared to patients in the low-Lp(a) group (55.0 ± 242.8 mm^3^ vs −25.0 ± 157.4 mm^3^; *P* = 0.020). In contrast, there was no difference in change in total plaque volume or change in calcific and noncalcific plaque volumes between the high- and low-Lp(a) groups.

**Table 2:** Change in Coronary Plaque Volumes on Repeat CCTA in Patients With High and Low Lp(a)

	Lp(a) ≥70 mg/dL (n = 36)	Lp (a) <70 mg/dL (n = 125)	*P* Value
Total plaque volume change	128.2 ± 330.6	88.5 ± 312.2	0.508
Calcific plaque volume change	19.7 ± 69.7	1.0 ± 85.7	0.231
Noncalcific plaque volume change	108.5 ± 319.6	87.5 ± 288.6	0.708
Low-density plaque volume change	26.2 ± 88.4	-0.7 ± 50.1	**0.020**
Fibro-fatty volume change	55.0 ± 242.8	-25.0 ± 157.4	**0.020**

Values are mean ± SD of the absolute difference in plaque volume (mm^3^) between scan 1 and 2. **Bold** *P* values <0.05 show statistically significant differences.

CCTA = coronary computed tomography angiography; Lp(a) = lipoprotein(a).

### Increasing Lp(a) is independently associated with progression of low-attenuation plaque

**Figure 3:**
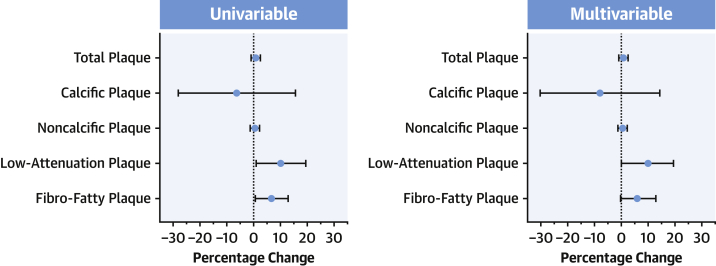
Effect of Lp(a) on Plaque Progression Data are depicted as betas with 95% CIs for the percentage change in plaque volume from baseline to follow-up CCTA, standardized for each 50 mg/dL increase in Lp(a). Lp(a) was associated with low-attenuation plaque progression in univariable (β = 10.2%, *P* = 0.031) and multivariable (β = 9.6%, *P* = 0.048) analyses, and with fibro-fatty plaque progression in univariable analysis (β = 6.7%, *P* = 0.034), showing a trend in multivariable analysis (β = 6.0%, *P* = 0.062). Abbreviations as in Figure 1.

In univariable regression analysis, Lp(a) was associated with progression of low-attenuation plaque volume (β = 11.6% for each 50 mg/dL increase, 95% CI: 2.0% to 21.2%; *P* = 0.018), but not with progression of total, calcific, and noncalcific plaque volumes (Figure 3). The association between high Lp(a) and accelerated progression of low-attenuation plaque volume remained in multivariable linear regression analysis adjusting for body mass index, ASSIGN score, and segment involvement score (β = 10.5% for each 50 mg/dL increase, 95% CI: 0.7% to 20.3%). Results were unchanged when considering high Lp(a) as a categorical variable (≥70 mg/dL) to evaluate its relation with absolute change in low-attenuation plaque volume (Table 3).

**Table 3:** High Lp(a) (≥70 mg/dL) and Absolute Change in Coronary Plaque Volumes on Repeat CCTA

	Univariable		Multivariable	
	Beta (95% CI)	*P* Value	Beta (95% CI)	*P* Value
Total plaque volume change	50.1 (−65.4 to 165.6)	0.393	−23.2 (−140.4 to 94.1)	0.697
Calcific plaque volume change	19.1 (−11.9 to 50.1)	0.226	−12.9 (−44.6 to 18.9)	0.425
Noncalcific plaque volume change	31.0 (−76.8 to 138.8)	0.571	−10.3 (−120.2 to 99.6)	0.853
Low-attenuation plaque volume change	27.9 (5.3 to 50.5)	**0.016**	25.4 (2.5 to 48.2)	**0.030**
Fibro-fatty plaque volume change	84.3 (17.6 to 150.9)	**0.014**	73.8 (6.3 to 141.3)	**0.032**

Values are betas with 95% CI for the absolute change in plaque volume (mm^3^) in patients with serum Lp(a) ≥70 mg/dL. Multivariable linear regression analysis is adjusted for body mass index, ASSIGN score, and segment involvement score. **Bold***P* values <0.05 show statistically significant differences.

Abbreviations as in Table 2.

Results were also similar regarding fibro-fatty plaque progression. Higher concentrations of Lp(a) were associated with accelerated progression of fibro-fatty plaque in univariable regression (β = 7.0% for each 50 mg/dL increase, 95% CI: 0.9% to 13.2%), and showed a trend in multivariable regression (β = 6.2% for each 50 mg/dL increase, 95% CI: −0.1 to 12.4%) (Tables 3 and 4).

**Table 4:** Increasing Lp(a) and Change in Coronary Plaque Volumes on Repeat CCTA

	Univariable		Multivariable	
	Beta (95% CI)	*P* Value	Beta (95% CI)	*P* Value
Total plaque volume change	0.3 (−3.9 to 4.5)	0.887	0.81 (−5.0 to 3.4)	0.701
Calcific plaque volume change	−9.6 (−40.6 to 21.4)	0.542	−12.75 (−44.5 to 19.0)	0.428
Noncalcific plaque volume change	0.0 (−4.2 to 4.3)	0.988	−1.0 (−5.2 to 3.2)	0.631
Low-attenuation plaque volume change	11.6 (2.0 to 21.2)	**0.018**	10.5 (0.7 to 20.3)	**0.037**
Fibro-fatty plaque volume change	7.0 (0.9 to 13.2)	**0.025**	6.2 (−0.1 to 12.4)	0.053

Values are betas with 95% CI for the percentage change in plaque volume, standardized for each 50 mg/dL increase in serum lipoprotein(a). Multivariable linear regression analysis is adjusted for body mass index, ASSIGN score and segment involvement score. **Bold***P* values <0.05 show statistically significant differences.

Abbreviations as in Tables 2 and 3.

## Discussion

For the first time, we have used repeat CCTA to investigate the association between serum Lp(a) concentrations and progression of coronary plaque volume and phenotype in patients with advanced multivessel coronary atherosclerosis. Patients with high serum Lp(a) concentrations (≥70 mg/dL) showed accelerated progression of low-attenuation coronary plaque volume after 12 months (Central Illustration). Conversely, we found no difference in the progression of more stable plaque phenotypes between the high- and low-Lp(a) groups. These findings provide a mechanistic explanation for the association between high Lp(a) and the residual risk of myocardial infarction in patients already established on secondary prevention.

**Central Illustration:**
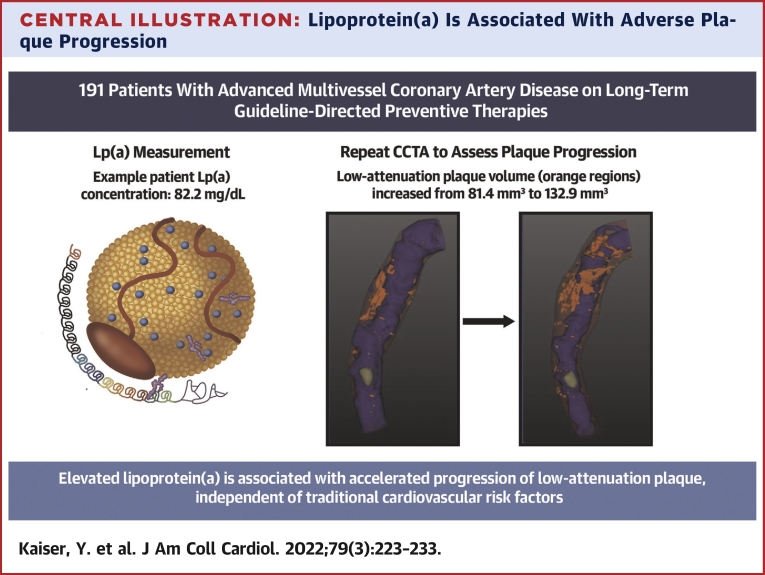
Lipoprotein(a) Is Associated With Adverse Plaque Progression Patients with advanced multivessel coronary artery disease with elevated Lp(a) concentrations (≥70 mg/dL) showed accelerated progression of low-attenuation plaque. An example patient with elevated Lp(a) (82.2 mg/dL) showed marked progression of low-attenuation plaque volume (visualized in **orange**): increased from 81.4 to 132.9 mm^3^ after 1-year follow-up. CCTA = coronary computed tomography angiography; Lp(a) = lipoprotein(a).

CCTA and semiautomated quantitative analysis techniques now allow for the noninvasive assessment of different plaque types and their progression over time. Adverse plaque phenotype, including a large necrotic core, inflammation, microcalcification, and a thin fibrous cap, are associated with an increased propensity to rupture and therefore with an increased risk of myocardial infarction (20). Low-attenuation plaque on CCTA is a quantitative marker of necrotic core and provides prognostic information for cardiovascular outcomes (22). Indeed, in the SCOT-HEART (Scottish Computed Tomography of the Heart) trial, the burden of low-attenuation plaque was the most powerful predictor of fatal or nonfatal myocardial infarction, outperforming cardiovascular risk scores, assessments of plaque lumen stenosis, CT calcium scoring, and the burden of all other different plaque subtypes (23). Similarly, powerful prognostic value was also reported in the recent ICONIC (Incident Coronary Syndromes Identified by Computed Tomography) study, a large prospective multinational registry of patients undergoing CCTA (11). In the current study, we found that Lp(a) was independently associated with accelerated progression of low-attenuation plaque in patients with comparable disease severity at baseline. These findings suggest that in established coronary artery disease, Lp(a) drives ASCVD-risk by promoting progression of vulnerable plaque phenotypes, providing a potential mechanistic explanation for the association between Lp(a) and clinical atherothrombotic events. This association appears robust and independent of baseline disease severity, cardiovascular risk factors, and plaque burdens. Moreover, this progression in low-attenuation plaque phenotype was observed despite the near universal prescription of statins and other preventative medications in this cohort. Therefore, our data provide further support for Lp(a) as a novel risk marker of residual risk as well as reaffirming its potential as a treatment target in coronary atherosclerosis, particularly in the population investigated here: patients with high Lp(a) already established on preventative therapies. Randomized controlled trials are underway investigating whether Lp(a)-lowering therapy can improve cardiovascular outcomes in this patient population. Our data pave the way for mechanistic substudies to investigate whether any observed clinical benefit is due to a slowing in low-attenuation plaque progression with Lp(a) lowering.

What do these findings imply for the pathophysiological role of Lp(a) in coronary artery disease? As we did not observe a difference between high- and low-Lp(a) groups in the progression of total plaque volume or more stable subtypes of plaque, the sheer accumulation of Lp(a) particles in the atherosclerotic plaque does not appear to be the dominant mechanism. Instead, we observed that patients with high Lp(a) showed accelerated progression of low-attenuation plaque phenotype, a marker for necrotic cores. Lp(a) and its associated OxPL are known to trigger a proinflammatory response, resulting in cellular apoptosis and necrosis thereby contributing to accelerated necrotic core formation (24,25). Blocking of OxPL signaling using an antibody treatment reduced necrotic core formation by almost one-half its size in a murine model of atherosclerosis (26,27). Furthermore, human pathology studies show the increasing presence of Lp(a) and OxPL in coronary artery plaques as lesion severity progresses (26,28). Increased levels of circulating Lp(a) may therefore drive low-attenuation plaque progression predominantly via OxPL and its proinflammatory effects.

We did not observe any baseline difference in coronary plaque burdens between patients with high and low Lp(a) concentrations. This is an important strength because it allows us to assess the effect of Lp(a) on plaque progression independent of baseline plaque burden, which is a major determinant of disease progression. However, it is seemingly at odds with several previous intravascular ultrasound studies that have shown baseline differences in plaque burden with Lp(a) concentrations (7,8). These cohorts consisted primarily of low-risk individuals in a primary prevention setting, whereas we have focused on patients with established multivessel disease and previous coronary revascularization, the majority of whom had had a previous acute coronary syndrome. These inclusion criteria are likely to have caused a selection pressure in our cohort resulting in the recruitment of patients who all have a high plaque burden and a high burden of adverse plaque irrespective of their Lp(a) concentrations.

### Study limitations

Our study includes one of the largest number of consecutive, prospectively enrolled patient cohorts to undergo repeat CCTA for the assessment of coronary plaque progression. However, we recognize that this is a single-center study comprising a largely Caucasian male population. Although we have previously shown the reproducibility of CCTA plaque assessments and repeat CCTA has been used in smaller randomized trials such as the EVAPORATE (Effect of Icosapent Ethyl on Progression of Coronary Atherosclerosis in Patients With Elevated Triglycerides on Statin Therapy) study, we acknowledge the need to confirm our findings in larger patient populations and cannot rule out smaller effects of Lp(a) on the progression of other coronary plaque subtypes (29,30). We have also focused on patients with advanced established coronary artery already established on secondary prevention, a common group in whom Lp(a) lowering is likely to prove the maximal benefit, and which is being investigated in the ongoing phase 3 Lp(a)-lowering Lp(a)HORIZON study (NCT04023552). Future studies across multiple centers using different scanners and enrolling more diverse populations with less advanced coronary artery disease are needed to explore the generalizability of our findings. Finally, quantification of plaque subtype can be a time-intensive process and the practical application of plaque quantification may be improved by further automation.

## Conclusions

High concentrations of serum Lp(a) are associated with accelerated progression of low-attenuation plaque (necrotic core) in patients with advanced multivessel coronary artery disease despite receiving guideline-based preventative therapies. This provides a potential mechanistic explanation for the association between Lp(a) and the residual risk of myocardial infarction and supports Lp(a) as a novel treatment target in atherosclerosis. Randomized controlled trials of Lp(a) lowering are now required to assess whether this reduces both low-attenuation plaque progression and cardiovascular events.

Perspectives **COMPETENCY IN MEDICAL KNOWLEDGE:** In patients with advanced coronary artery disease, elevated serum Lp(a) is associated with accelerated progression of the low-attenuation necrotic core of atherosclerotic plaques.

**TRANSLATIONAL OUTLOOK:** Randomized trials are required to evaluate whether Lp(a) lowering reduces low-attenuation plaque progression and prevents ischemic events.

## Funding Support and Author Disclosures

Drs Kaiser and Stroes were supported by the Netherlands Heart Foundation CVON 2017-20: generating the best evidence-based pharmaceutical targets for atherosclerosis [GENIUS II]). Dr Dey is supported by the National Institute of Health/National Heart, Lung, and Blood Institute grants (1R01HL148787-01A1 and 1R01HL151266). Dr Dweek is supported by the British Heart Foundation (FS/14/78/31020) and is the recipient of the Sir Jules Thorn Award for Biomedical Research 2015 (15/JTA); has received speaker fees from Pfizer and Novartis; and has received consultancy fees from Novartis, Jupiter Bioventures, and Silence therapeutics. Dr Stroes has received research grants/support to his institution from Amgen, Sanofi, Resverlogix, and Athera; and has served as a consultant for Amgen, Sanofi, Esperion, Novartis, and Ionis Pharmaceuticals. All other authors have reported that they have no relationships relevant to the contents of this paper to disclose.
